# Human Umbilical Cord Mesenchymal Stem Cell-Derived Extracellular Vesicles Carrying MicroRNA-29a Improves Ovarian Function of Mice with Primary Ovarian Insufficiency by Targeting HMG-Box Transcription Factor/Wnt/*β*-Catenin Signaling

**DOI:** 10.1155/2022/5045873

**Published:** 2022-07-02

**Authors:** Tian Gao, Yi Cao, Min Hu, Ying Du

**Affiliations:** ^1^Department of Gynecology and Obstetrics, The First Affiliated Hospital of Chongqing Medical University, Chongqing 400016, China; ^2^Reproductive Medicine Center, The First Affiliated Hospital of Chongqing Medical University, Chongqing 400016, China

## Abstract

**Background:**

Primary ovarian insufficiency (POI) is a female disease characterized by ovarian function loss under 40 years old. Transplantation of exosomes is an encouraging regenerative medicine method that has the potential for restoring ovarian functions post-POI with high efficiency. Therefore, we investigate the therapeutic efficacy and potential mechanisms of human umbilical cord mesenchymal stem cell- (UCMSC-) derived exosomes on ovarian dysfunction post-POI.

**Methods:**

The model of POI was established by intraperitoneal injection with 5 mg/kg cisplatin. The mouse ovarian function was detected by measuring the levels of anti-Mullerian hormone, follicle-stimulating hormone, and estradiol and detecting the morphological changes. For in vitro experiments, the characterization and identification of UCMSCs and UCMSC-derived exosomes were done by observation of morphologies and flow cytometry. To exclude the interference effect of nonspecific precipitation substances, UCMSCs were treated with RNase A or RNase A in combination with Triton X-100. Granulosa cell (GC) identification was performed using immunofluorescence. GC proliferation and viability were assessed using 5-ethynyl-2′-deoxyuridine (EdU) assays and Cell Counting Kit-8 (CCK-8), and GC apoptosis was calculated by flow cytometry. Gene expression and protein levels were evaluated using reverse transcription quantitative polymerase chain reaction (RT-qPCR) and western blotting. The binding relationship between miR-29a and HMG-box transcription factor (HBP1) was verified by luciferase reporter assays.

**Results:**

In vitro, the human UCMSC-derived exosomes carrying miR-29a upregulation promoted the proliferation of GCs and suppressed their apoptosis. In vivo, miR-29a upregulation reserved the mature follicles and restored the ovarian functions. miR-29a targeted HBP1 and negatively regulated its expression. HBP1 upregulation rescued the miR-29a upregulation-induced inhibition in GC apoptosis and inactivated the Wnt/*β*-catenin pathway.

**Conclusion:**

The exosomal miR-29a derived from human UCMSCs improves the ovarian function by targeting HBP1 and activating the Wnt/*β*-catenin pathway.

## 1. Introduction

Primary ovarian insufficiency (POI) is a complicated endocrine disease [1]. A meta-analysis of the global prevalence of POI finds a rate of 3.7% [2]. POI is usually accompanied by psychological distress, osteoporosis, diabetes, metabolic syndrome, and ischemic heart disease [3]. Follicle dysfunction or depletion is considered the most important mechanisms in POI [4]. Genetic and autoimmune conditions are two main contributors of POI pathogenesis [5]. The diagnostic methods of POI include the measurement of anti-Mullerian hormone (AMH), inhibin B, follicle-stimulating hormone (FSH), and estradiol (E_2_) [6]. The concentration of AMH is gradually decreased as the age and size of follicle increase [7]. FSH works together with luteinizing hormone (LH) to facilitate follicular maturation, estrogen secretion, and ovulation [8]. This disorder is mainly characterized by declining ovarian functions, which are related to amenorrhea, high gonadotropin, and low estrogen levels [9]. Currently, the primary treatment modality for patients with POI is hormone replacement therapy (HRT) which can alleviate vasomotor and urogenital symptoms and prevent cardiovascular diseases and osteoporosis. However, the optimal dosage, hormone preparations used, and duration of HRT for patients have not been fully investigated [10]. Therefore, a better understanding of POI mechanisms is of great significance to initiate effective therapeutic strategies.

Undifferentiated cells, called stem cells, are characterized by the abilities of self-renewal and regeneration and considered effective options for POI treatment [11]. Stem cells include mesenchymal stem cells (MSCs), induced pluripotent stem cells (iPSCs), stem cells from extraembryonic tissues, and ovarian stem cells [12]. MSCs belong to the family of nonhematopoietic stem cells [13]. Isolation of MSCs from umbilical cord blood, bone, and adipose tissues has been reported [14]. The mouse umbilical cord-derived MSCs (UCMSCs) were applied in POI treatment in 2013. During this treatment, the researchers discovered the reduction in cell apoptosis, elevation in the sex hormone level, and recuperation of ovarian functions [15]. The paracrine effectors have been reported to mediate the therapeutic effects of MSCs [16]. As major paracrine effectors, extracellular vesicles (EVs) play a critical role in transferring bioactive component. Exosome, a member of EVs, is involved in intermolecular interaction by delivering multiple self-carrying proteins and noncoding RNAs [17]. Human UCMSCs have the advantages of wide source, convenient collection, and low immunogenicity [18]. A previous study has proven that transplantation of human UCMSCs can protect ovarian granulosa cells (GCs) from death and mediate the immune factors to administer POI [19]. However, the underlying mechanisms of exosomes against ovarian functions have not been fully explored.

Exosomes mediate local cell-to-cell communication by transferring mRNA, lncRNA, microRNA, proteins, and lipids [20]. MicroRNAs (miRNAs) with 18-25 nucleotides in length are small noncoding RNAs [21]. Recent studies have revealed that miRNAs carried by exosomes exert functions in POI progression. For example, miR-644-5p, an MSC-derived exosome, inhibits the apoptosis of GCs and has the potential to restore ovarian functions [22]. Exosomal miR-144-5p alleviates the chemotherapy-induced POI in rats [23]. Exosomes reduce GC apoptosis via transferring miR-1246 [24]. Moreover, exosomes carrying miR-146a and miR-10a protect chemotherapy-treated mice from ovarian follicular atresia [25]. As reported, miR-29a level is found to be lower in POI tissues than in normal tissue samples, and downregulation of miR-29a is positively correlated with high hormone levels in POI ovarian tissues [26]. However, specific functions of miR-29a and its mechanisms against POI progression remain unclear.

The HMG-box transcription factor 1 (HBP1) is a member of the sequence-specific HMG family [27]. The Wnt pathway consists of the Wnt family secreted protein, the Frizzled family, and *β*-catenin. In intestinal fibrosis, HBP1 downregulation promotes the activation of Wnt/*β*-catenin [28]. Accumulating evidence has indicated that the Wnt/*β*-catenin pathway participates in the regulation of ovarian functions. For example, the Wn4 depletion in mice leads to poor ovarian development and induces the apoptosis of healthy follicles [29]. The normal *β*-catenin expression in ovarian tissues can facilitate the secretion of FSH, thereby resulting in the promotion in follicular development and reduction in GC apoptosis [30, 31].

In the current study, we identified the presence of miR-29a in human UCMSC-derived exosomes. We purposed to explore the biological functions of miR-29a carried by the human UCMSC-derived EVs and its mechanisms against ovarian functions. We hypothesized that exosomal miR-29a might play a protective effect in POI. We believe that exosomal miR-29a would serve as a promising target for POI treatment.

## 2. Methods

### 2.1. Isolation of Human UCMSCs

Human umbilical cords obtained from the parturient women were used for our study, and the protocols were performed as previously described [32]. All participants provided the written and informed consent. After washing, the umbilical cords were cut into blood vessel-free pieces (1 mm^3^). Then, the small pieces were incubated in Dulbecco's modified Eagle's medium (DMEM; PM150210B, Procell, Wuhan, Hubei, China) at 37°C with 5% CO_2_. A light microscope (Imager.D2; ZEISS, Germany) was used for morphology observation. The Alizarin Red staining kit (ZY121105, Zeye Biotech, Shanghai, China) was prepared for identification of osteogenic differentiation, and the Oil Red O staining (E607319, Sangon Biotech, Shanghai, China) was utilized to identify adipogenic differentiation. The molecular markers, including CD29, CD44, CD90, CD14, CD34, and HLA-DR, were detected by flow cytometry (FACSCanto II, BD, San Diego, CA, USA).

### 2.2. Human UCMSC-Derived Exosomes

When the isolated human UCMSCs at the fourth to sixth passage incubated in DMEM with 10% fetal bovine serum (FBS; FSP100, ExCell Biotech, Shanghai, China) reached 70%-80% confluence, the medium was centrifuged at 2,000 × *g* for 15 min at 4°C and then filtered with a 0.2 mm filter. Subsequently, the filtered medium was ultracentrifuged at 10,000 × *g* at 4°C. To remove the supernatant, it was ultracentrifuged at 100,000 × *g* for 20 min. After washing using the phosphate-buffered saline (PBS; P1022, Solarbio, Beijing, China), the exosomes derived from the human UCMSCs were stored at -80°C. The identification of exosomes was performed by measuring the surface marker using western blotting [33].

### 2.3. Cell Transfection

The miR-29a mimics (50 nM) as well as the HBP1 overexpression vector pcDNA3.1-HBP1 (4 *μ*g) and their corresponding negative controls were purchased from GeneChem (Shanghai, China). Human UCMSCs seeded into six-well plates (2 × 10^5^ cells/well) were transfected with above plasmids using Lipofectamine 2000 (L3287, Sigma-Aldrich, St. Louis, MO, USA), and then, the exosomes were isolated from UCMSCs for the subsequent experiments. The transfection efficiency was detected using RT-qPCR after 24 h of transfection.

### 2.4. Mouse Ovarian GCs

The animal experiments were granted approval of the Ethical Committee of the First Affiliated Hospital of Chongqing Medical University (Chongqing, China), and the experimental protocol was under the National Research Council Guide for Care and Use of Laboratory Animals. C57BL/6 mice (3 weeks old, female, healthy) were obtained from the GemPharmatech (Nanjing, Jiangsu, China). The mice were under standard housing of 25 ± 1°C with a humidity of 50 ± 5% and kept in a 12/12 h light/dark cycle. Standard food and water were available to the mice. Premature GCs were isolated in accordance with the previous method [25]. The intraperitoneal injection of pregnant mare serum gonadotropin (PMSG; P9970, Solarbio) induced the stimulation of follicle growth. After 48 h of PMSG injection, the mice were euthanized, and the ovaries were obtained. Then, GC isolation was done by puncturing the preovulatory follicles using 30-guage needles under the anatomical microscope (SMZ-10A, Nikon, Tokyo, Japan). The isolated cells were washed using PBS and cultured in DMEM supplemented with 10% FBS, 100 U/ml penicillin, and 100 *μ*g/ml streptomycin in an incubator (37°C, 5% CO_2_, humidified atmosphere). GCs at the first passage were used in the experiments. For immunofluorescence staining, the GCs were incubated with a primary antibody against FSHR (FNab03231, Fine Biotech, Wuhan, Hubei, China) for 12 h at 4°C and then incubated with the secondary antibody for 30 min at 37°C. The examination of the signals from each sample was conducted using a fluorescence microscope (AxioCam HRc; ZEISS) following the DAPI staining (ab228549, Abcam).

### 2.5. UCMSC and GC Transwell Coculture

To investigate the biological functions of HUCMSC-derived exosomes carrying miR-29a on ovarian functions of mice, we performed UCMSC and GC transwell coculture as previously described [34]. Human UCMSCs plated onto 24-well transwell permeable support (1 × 10^5^ cells/well; pore size, 0.4 *μ*m; 3391, Sanger, Shanghai, China) after indicated treatment as described above were cultured overnight at 37°C with 5% CO_2_. Then, 5 × 10^4^ cells/well of GCs were seeded into the bottom of 24-well plates, and they were cocultured for 24 h.

### 2.6. Animal Model of POI Establishment

An animal model of POI was established as previously described [35]. C57BL/6 mice (eight weeks old, female, healthy) were obtained from the GemPharmatech. The mice were housed in an air-conditioned facility with a 12 h light/dark cycle at 25 ± 1°C and 50 ± 5% humidity and supplied with free food and water. The mice were grouped into the sham+Exos-miR-NC group, the sham+Exos-miR-29a group, the POI+Exos-miR-NC group, and the POI+Exos-miR-29a group (*n* = 8). The mice in the sham groups were injected intraperitoneally with normal saline and then exosomes (125 *μ*g dissolved in 100 *μ*l PBS) carrying miR-29a mimics or miR-NC into the tail vein. The mice in the POI groups were injected intraperitoneally with 5 mg/kg cisplatin and then exosomes carrying miR-29a mimics or miR-NC into the tail vein. After 15 d, the mice were sacrificed, and the ovarian tissues and blood were taken for the subsequent experiments.

### 2.7. Reverse Transcription Quantitative Polymerase Chain Reaction (RT-qPCR)

The RT-qPCR was conducted based on the methods reported [22]. After total RNA extraction using TRIzol reagent (Sigma-Aldrich), total RNA (1 *μ*g) was reverse transcribed into cDNA using the FastQuant RT Kit (with gDNase; TAG200, Biolab, Beijing, China). RT-qPCR was conducted using SYBR Green (Solarbio). The miRNA and mRNA expression levels were calculated using the 2^−△△CT^ method. U6 and GAPDH were used as the internal references for miRNAs and mRNAs, respectively. The premier sequences used in this study are listed in Table 1.

### 2.8. Western Blotting

Western blotting was performed according to a previous method [36]. Protein isolation from mouse ovaries and GCs was performed using the RIPA lysis buffer (R0020, Solarbio). A BCA protein assay kit (PC0020, Solarbio) was used to measure the protein concentration. The protein samples were denatured at 100°C for 10 min. Then, the proteins were separated by sodium sulfate-polyacrylamide gel electrophoresis (SDS-PAGE) and transferred onto polyvinylidene difluoride (PVDF) membranes. The membranes subsequently were blocked using 5% skimmed milk for 1 h at room temperature. After blocking, the membranes were incubated overnight with primary antibodies against CD63 (ab134045, 1 : 1000; Abcam), calnexin (ab133615, 1 : 1000; Abcam), HBP1 (ab216844, 1 : 1000; Abcam), T cell factor (TCF; ab272235, 1 : 1000; Abcam), lymphoid enhancer factor (LEF; ab137872, 1 : 1000; Abcam), p-GSK3*β* (ab68476, 1 : 1000; Abcam), histone H3 (ab1791, 1 : 1000; Abcam), GSK3*β* (ab185141, 1 : 5000; Abcam), *β*-catenin (ab184919, 1 : 1000; Abcam), and GAPDH (ab9485, 1 : 2500; Abcam). The next day, the membranes were washed three times using Tris-buffered saline Tween-20 (Sigma-Aldrich) and incubated with secondary antibodies at room temperature for 1 h. The protein level was detected using the ChemiDoc MP Imaging System (Bio-Rad, Hercules, CA, USA).

### 2.9. 5-Ethynyl-2′-deoxyuridine (EdU) Assay

The GC cells seeded in 96-well plates (5 × 10^3^ cells/well) were incubated for 24 h. Cell proliferation was assessed using the EdU Imaging Kits (BN16015, BioDee, Beijing, China). A fluorescence microscope was utilized for the observation of EdU-positive cells.

### 2.10. Cell Counting Kit-8 (CCK-8)

The 96-well plates (5 × 10^3^ cells/well) were applied for GC incubation. Then, the GCs were cocultured with the supernatant of UCMSCs with indicated transfection for 24 h. Next, 10 *μ*l of CCK-8 reagent (40203ES60, Yeasen, Shanghai, China) was added. After 2 h incubation at 37°C, the absorbance at 450 nm was measured using a microplate reader (Molecular Devices, Shanghai, China).

### 2.11. Cell Apoptosis Analysis

GCs were seeded in six-well plates at a density of 5 × 10^5^ cells/well and incubated for 48 h. After washing using precooled PBS twice, the GCs were stained with FITC-Annexin V and propidium iodide (PI) (Solarbio) for 15 min at room temperature using the Annexin V-FITC Apoptosis Detection Kit (CA1020, Solarbio). The stained cells were analyzed using flow cytometry.

### 2.12. Luciferase Reporter Assay

The wild-type (WT) 3′ untranslated region (3′UTR) of HBP1 was purchased from (Genomeditech, Shanghai, China) and was subcloned in the pGL3-Baise luciferase reporter vector (VT1554, Youbio, Hunan, China). The mutant sequences (Mut) were obtained by QuikChange mutagenesis (200523, Agilent, Beijing, China). Then, the Wt or Mut vector was cotransfected with miR-29a mimics into UCMSCs using Lipofectamine 2000. After 24 h, the luciferase activity was determined using a dual-luciferase reporter assay kit (DL101-01, Vazyme, Nanjing, China).

### 2.13. RNA Pull-Down Assays

RNA pull-down assays were performed as previously described [37]. The cells were transfected with biotinylated miR-29a mimics or biotin-negative control (50 nM) for 48 h and harvested after transfection. Then, the cells were lysed using lysis buffer (Ambion Company, Austin, TX, USA) for 10 min. The lysate was incubated with streptavidin magnetic beads (11206D, Invitrogen, Carlsbad, CA, USA) at 4°C overnight. After washing twice with precooled lysis buffer, thrice with low salt buffer, and once with high salt buffer in successive, the bound RNA was purified using TRIzol and the enrichment was measured by RT-qPCR analysis.

### 2.14. Hematoxylin and Eosin (HE) Staining

Ovarian tissues were fixed with 4% formaldehyde for 24 h, embedded in paraffin, and serially cut into 5 *μ*m sections. The sections were then stained with hematoxylin and eosin (HE; E607318-0200, Sangon) to estimate the follicle growth. The morphological characteristics of ovarian sections were observed using light microscopy. Follicle counts were conducted on serially cut sections, counting every tenth section for each animal. The mean count per section was calculated. Follicle stages were classified as described previously [38].

### 2.15. Measurement of AMH, E_2_, and FSH

The serum was obtained by centrifugating the blood samples of mice taken from postcava at 5000 rpm for 10 min. AMH, E_2_, and FSH levels in serum were measured using the ELISA kit (Enzyme-Linked Biotechnology, Shanghai, China) according to the manufacturer's instructions.

### 2.16. Statistical Analysis

Data was analyzed using GraphPad Prism 6.0 software (GraphPad Inc., San-Diego, CA, USA) and expressed as the mean ± standard deviation. Student's *t*-test was conducted to compare differences between groups. The comparisons among multiple groups were analyzed using the one-way analysis of variance followed by Tukey's *post hoc* analysis. *p* < 0.05 is considered statistically significant.

## 3. Results

POI is a female disease characterized by ovarian function loss under 40 years old. Transplantation of human UCMSCs can protect ovarian granulosa cells (GCs) from death and mediate the immune factors to administer POI, and human UCMSC-derived exosomes can promote the production of estrogen (estrogen deficiency is a clinical feature of POI). miR-29a level is found to be lower in POI tissues than in normal tissue samples, and downregulation of miR-29a is positively correlated with high hormone levels in POI ovarian tissues. Therefore, in the current study, we purposed to investigate the biological functions of miR-29a carried by the human UCMSC-derived EVs and its associated mechanisms involved in the regulation of ovarian functions.

### 3.1. Characterization and Identification of UCMSCs and UCMSC-Derived Exosomes

**Figure 1:**
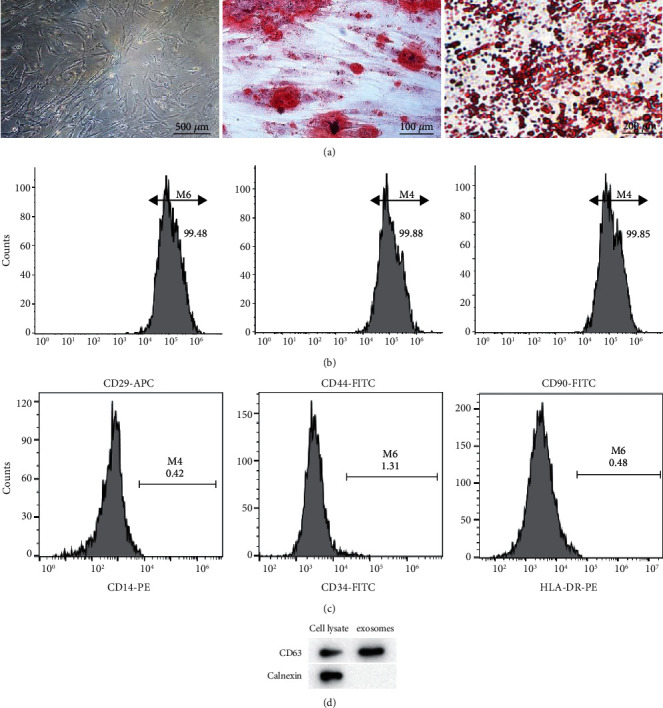
Identification of UCMSCs and characterization of exosomes derived from UCMSCs. (a) The morphology of human UCMSCs at the fourth passage, the Alizarin Red staining of osteogenic identification, and the Oil Red O staining of adiopogenic differentiation. (b, c) Positive expression of CD29, CD44, and CD90 and negative expression of CD14, CD34, and HLA-D-PE were detected by flow cytometry. (d) Western blotting was performed to measure the protein level of positive exosome marker (CD63) and negative exosome marker (calnexin).

The extracted UCMSCs were identified via the detection of the pluripotent differentiation potential and surface markers of cells. As shown in Figure 1(a), human UCMSCs displayed fibroblast-like morphology. In the osteoblastic induction medium, the calcium nodules were formed, and the radioactive center was orange red following the Alizarin Red staining. After the adipogenic induction and Oil Red O staining, the lipid droplets in the cytoplasm were positive. The positive expression of CD29, CD44, and CD90, as well as the negative expression of CD14, CD34, and HLA-DR, was demonstrated by flow cytometry (Figures 1(b) and 1(c)). As western blotting showed, the protein level of CD63 (exosome surface marker) was upregulated compared with that of cell lysate, while calnexin (intracellular protein) was absent in the isolated exosomes (Figure 1(d)). These results indicate that exosomes have been isolated from human UCMSCs.

### 3.2. UCMSC-Exosomes Can Deliver miR-29a into the GCs

**Figure 2:**
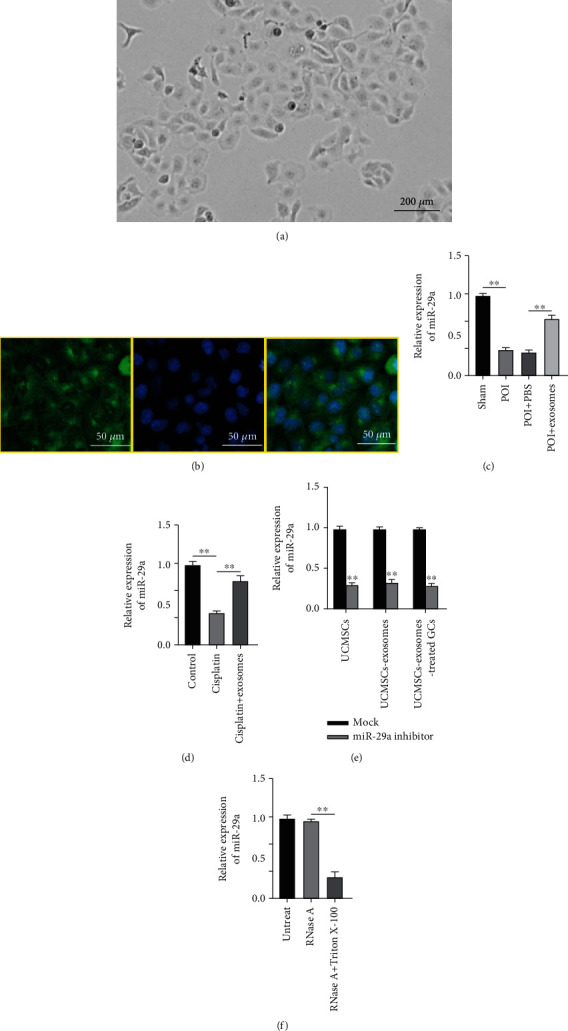
UCMSC-exosomes can deliver miR-29a into the GCs. (a) Morphology of isolated GCs. (b) Immunofluorescence of GC surface marker (FSHR). (c) miR-29a level in the sham, the POI, the POI+PBS, and the POI+exosome groups. (d) miR-29a level in the control, the cisplatin, and the cisplatin+exosome groups. (e) miR-29a level in UCMSCs, UCMSC-exosomes, and UCMSC-exosome-treated GCs. (f) UCMSCs were treated with RNase A or RNase A in combination with Triton X-100. RT-qPCR was conducted to measure the miR-29a level. ^∗∗^*p* < 0.01.

The isolated GCs from mouse ovarian tissues were incubated in vitro, and the GCs exhibited polygon-like morphology after 48 h of initial plating (Figure 2(a)). FSHR is a molecular marker for identification of GCs. The green fluorescence revealed that they are GCs, as revealed by immunofluorescence staining (Figure 2(b)). Lower miR-29a level was observed in the POF group than in the sham group, while exosome injection attenuated the inhibitory effects of POF insult on miR-29a level (Figure 2(c)). In cisplatin-challenged GCs, miR-29a level was downregulated. However, exosome addition rescued the decreased miR-29a level (Figure 2(d)). To confirm whether UCMSC-exosomes delivered miR-29a into the GCs, we effectively knocked down miR-29a level in UCMSCs. Subsequently, reduced miR-29a level was also observed in the UCMSC-exosomes and UCMSC-exosome-treated GCs (Figure 2(e)). Finally, the cells were treated using RNase A or RNase A in combination with Triton X-100 to exclude the interference effect of nonspecific precipitation substances, and the results ensured that miR-29a was wrapped by the UCMSC-exosomes (Figure 2(f)). These results demonstrated that UCMSC-exosomes can deliver miR-29a into the GCs.

### 3.3. Exosomes Carrying miR-29a Promotes the Proliferation and Inhibits the Apoptosis of GCs

**Figure 3:**
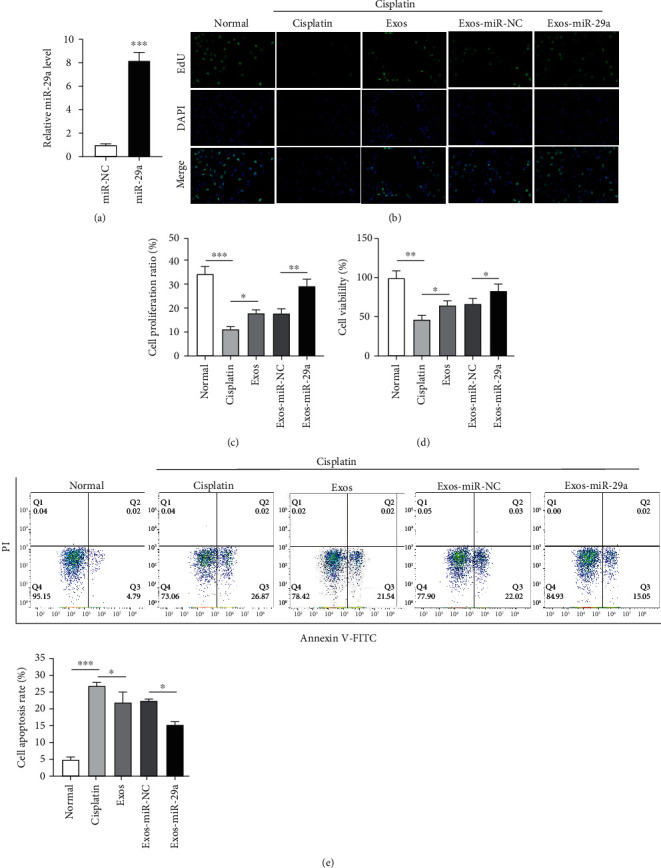
Exosomes carrying miR-29a promote proliferation and inhibit apoptosis of GCs. (a) The overexpression efficiency of miR-29a mimics detected by RT-qPCR. (b) EdU assays of GC proliferation in the normal group, the cisplatin group, the Exos group, the Exos-miR-NC group, and the Exso-miR-29a group. (c) Proliferation ratio of GCs. (d) CCK-8 of GC viability after indicated treatment. (e) Flow cytometry of GC apoptosis. ^∗^*p* < 0.05, ^∗∗^*p* < 0.01, and ^∗∗∗^*p* < 0.001.

Currently, exosomes have been reported to carry miRNAs [39]. A previous study has revealed the lower level of miR-29a in POI tissues than in normal tissue samples. We effectively overexpressed miR-29a level in UCMSCs by transfection with miR-29a mimics, and the overexpression efficiency was detected by RT-qPCR (Figure 3(a)). As EdU assays revealed, the cisplatin-induced decreased proliferation of GCs was rescued by exosomes, and miR-29a upregulation strengthened the promoting effect of exosomes (Figures 3(b) and 3(c)). Consistent with this finding, miR-29a upregulation enhanced the exosome-induced restoration in GC viability, as shown by CCK-8 assays (Figure 3(d)). Furthermore, flow cytometry was conducted to assess GC apoptosis, and the results demonstrated that exosomes restored the cisplatin-induced promotion in GC apoptosis and miR-29a upregulation further intensified the inhibitory effect of exosomes (Figure 3(e)). These results show that the exosomal miR-29a derived from human UCMSCs protects GCs against cisplatin-induced cell apoptosis.

### 3.4. miR-29a Targets HBP1 3′UTR

**Figure 4:**
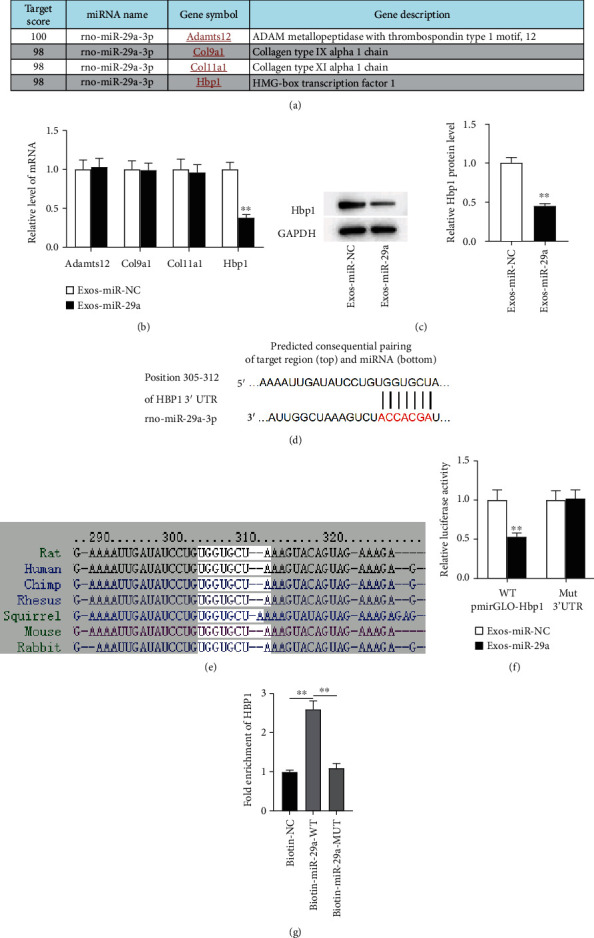
miR-29a targets HBP1 3′UTR. (a) miRDB of downstream target prediction (target score ≥ 98) (http://mirdb.org/). (b) RT-qPCR was carried out to measure the levels of candidate mRNAs in the context of miR-29a upregulation. (c) The protein level of HBP1 after miR-29a upregulation was determined by western blotting. (d) TargetScan of binding site prediction (http://www.targetscan.org/vert_80/). (e) The binding sites between miR-29a and HBP1 among species. (f) Luciferase reporter assays were conducted to verify the binding relationship of miR-29a and HBP1. (g) The relationship between miR-29a and HBP1 was tested by RNA pull-down assays. ^∗∗^*p* < 0.01.

To explore the underlying mechanism of miR-29a, miRDB was used to predict the downstream targets of miR-29a (screening criterion: target score ≥ 98), and Adamts12, Col9a1, Col11a1, and HBP1 were selected as candidate targets (Figure 4(a)). As RT-qPCR revealed, the mRNA level of HBP1 was downregulated in human UCMSCs after miR-29a upregulation, while no significant changes had been found in other groups (Figure 4(b)). Therefore, HBP1 was selected for our study. The protein level of HBP1 was decreased following miR-29a upregulation, as revealed by western blotting (Figure 4(c)). The binding sites between miR-29a and HBP1 are predicted by TargetScan, and the sites are highly conserved among species (Figures 4(d) and 4(e)). To verify the binding relationship between miR-29a and HBP1, luciferase reporter assays were conducted, and the results showed that miR-29a upregulation inhibited the luciferase activity of the wild-type HBP1 3′UTR, while miR-29a upregulation exerted no obvious effects on the luciferase activity of mutant HBP1 3′UTR (Figure 4(f)). The results of RNA pull-down assays showed that HBP1 RNA would be pulled down by bio-miR-29a-WT, whereas the corresponding bio-miR-29a-MUT had no significant effect on HBP1 expression (Figure 4(g)). These findings indicate that miR-29a targets HBP1 3′UTR.

### 3.5. HBP1 Upregulation Attenuates the Inhibitory Effects of miR-29a Upregulation on Cell Apoptosis

**Figure 5:**
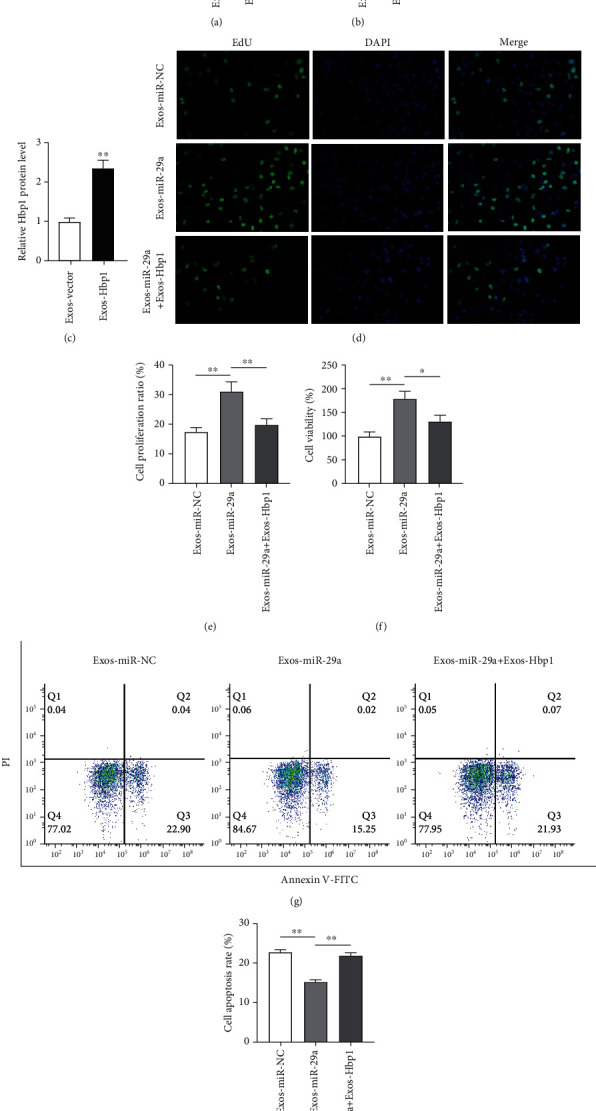
HBP1 upregulation attenuates the inhibitory effects of miR-29a upregulation on the apoptosis of GC. (a–c) Overexpression efficiency of pcDNA3.1-HBP1 was identified by RT-qPCR and western blotting. (d, e) EdU of GC proliferation in the Exos-miR-NC group, the Exos-miR-29a group, and the Exos-miR-29a+Exos-HBP1 group. (f) CCK-8 of cell viability. (g, h) Flow cytometry of cell apoptosis. ^∗^*p* < 0.05, ^∗∗^*p* < 0.01, and ^∗∗∗^*p* < 0.001.

To identify whether the miRNA-mRNA network exerted functions in GCs, we effectively upregulated HBP1 level using the pcDNA3.1-HBP1 and conducted RT-qPCR and western blotting to detect the overexpression efficiency (Figures 5(a)–5(c)). As EdU assays revealed, HBP1 restored the promotion in GC proliferation induced by miR-29a upregulation (Figures 5(d) and 5(e)). The miR-29a upregulation-induced increased viability of GCs was inhibited following HBP1 elevation, as shown by CCK-8 (Figure 5(f)). miR-29a inhibited cell apoptosis, while HBP1 played an opposite role in GC apoptosis, as shown by flow cytometry (Figures 5(g) and 5(h)). These results indicate that HBP1 restores the miR-29a upregulation-induced inhibition in GC apoptosis.

### 3.6. miR-29a Regulates the Wnt/*β*-Catenin Pathway by Targeting HBP1

**Figure 6:**
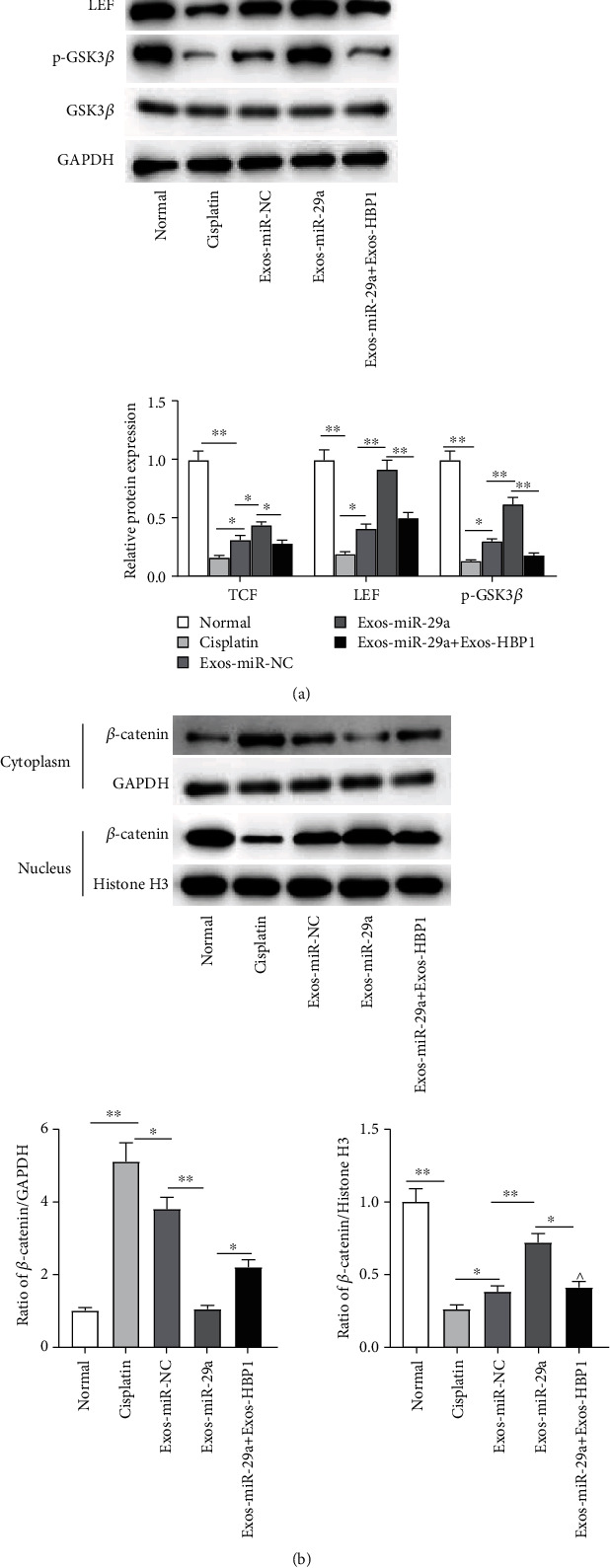
miR-29a regulates the Wnt/*β*-catenin pathway by targeting HBP1. (a) Protein levels of TCF, LEF, and phosphorylated GSK3*β* were measured by western blotting. (b) The cytoplasmic and nuclear fraction lysates were used to analyze the translocation of *β*-catenin by western blotting. ^∗^*p* < 0.05, ^∗∗^*p* < 0.01.

Then, we explore the mechanisms by which miR-29a and HBP1 mediate GC apoptosis. As reported, HBP1 leads to the inactivation of the Wnt/*β*-catenin pathway. Therefore, we performed western blotting to measure the levels of pathway-associated proteins, and the results showed that miR-29a upregulation reversed the cisplatin-induced inhibitory effects on protein levels of TCF, LEF, and phosphorylated GSK3*β*, while HBP1 upregulation restored the promoting effects of miR-29a upregulation (Figure 6(a)). Additionally, miR-29a upregulation resulted in the nuclear distribution of *β*-catenin, while HBP1 upregulation caused *β*-catenin transferring to the cytoplasm (Figure 6(b)). These results show that miR-29a regulates the Wnt/*β*-catenin pathway by targeting HBP1.

### 3.7. The Exosome Carrying miR-29a Restores the Ovarian Function *In Vivo*

**Figure 7:**
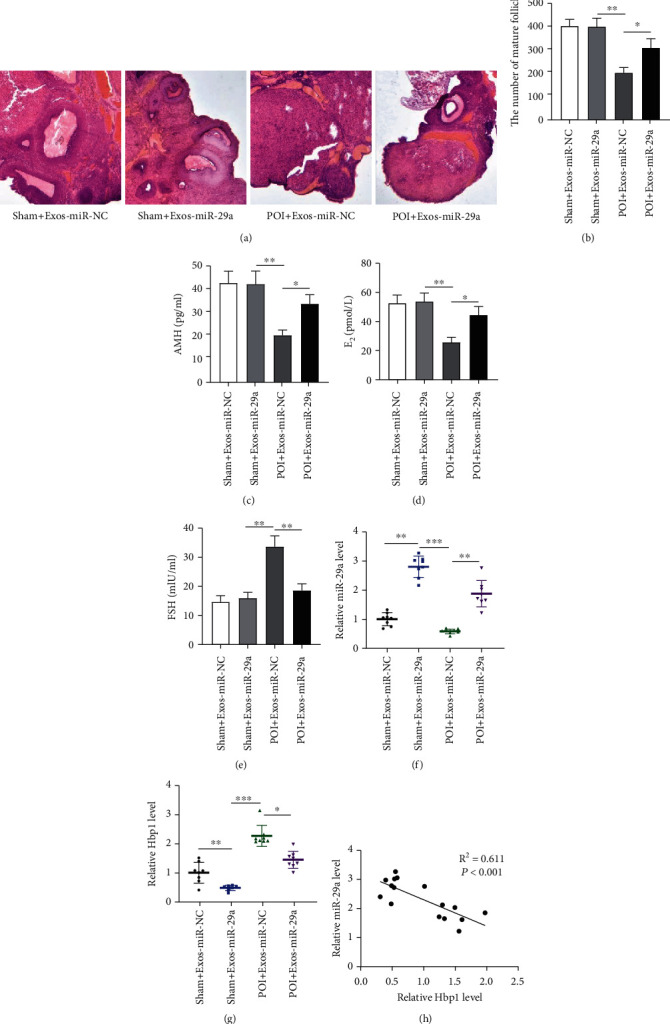
The exosome carrying miR-29a improves the ovarian function *in vivo*. (a) H&E staining was performed to examine the histopathological changes of ovarian tissues in the sham+Exos-miR-NC group, the sham+Exos-miR-29a group, the POI+Exos-miR-NC group, and POI+Exos-miR-29a group. (b) Quantification on follicle count in different groups. (c–e) The levels of AMH, E_2_, and FSH in indicated groups. (f, g) The levels of miR-29a and HBP1 in ovarian tissues. (h) The correlation of miR-29a and HBP1. ^∗^*p* < 0.05, ^∗∗^*p* < 0.01, and ^∗∗∗^*p* < 0.001.

To explore the therapeutic effects of human UCMSC-derived exosomes on restoration of ovarian function, an *in vivo* model of POF was established by intraperitoneal injection of 5 mg/kg cisplatin. The ovarian tissue of sham-operated mice stained with H&E showed large and abundant follicles, follicular fluid, and corpus luteum. The follicles in the POF group were few, and the atresia follicles formed by granule cell damage and the interstitial increased. However, exosomal miR-29a upregulation decreased the atresia follicles and increased the corpus luteum (Figure 7(a)). After collection of ovaries, the follicle number was counted. The results demonstrated that miR-29a upregulation rescued the POF-induced reduction in the number of mature follicles (Figure 7(b)). Then, we measured the levels of AMH, E_2_, and FSH in the serum of POI models and found that compared with those in the sham groups, the levels of AMH and E_2_ were decreased in the POI groups, and miR-29a upregulation attenuated the inhibitory effects of POI insult on AMH and E_2_ levels (Figures 7(c) and 7(d)). Opposingly, the FSH level was higher in the POI groups than in the sham groups, while miR-29a upregulation restored the promotion (Figure 7(e)). Finally, the levels of miR-29a and HBP1 in the ovarian tissues were detected. The results demonstrated that compared with that in the sham+Exos-miR-NC group, the miR-29a level was downregulated in the POI+Exos-miR-NC group, and miR-29a mimics significantly upregulated miR-29a level in ovarian tissues (Figure 7(f)). For HBP1, its level was downregulated in the context of miR-29a upregulation (Figure 7(g)).  Figure 7(h) presents the negative correlation between miR-29a and HBP1. These results show that the human UCMSC-derived exosome carrying miR-29a alleviates the POI-induced ovarian dysfunctions *in vivo* by targeting HBP1.

### 3.8. The Proposed Mechanism of miR-29a-Mediated Cell Proliferation through the Wnt/*β*-Catenin Pathway

**Figure 8:**
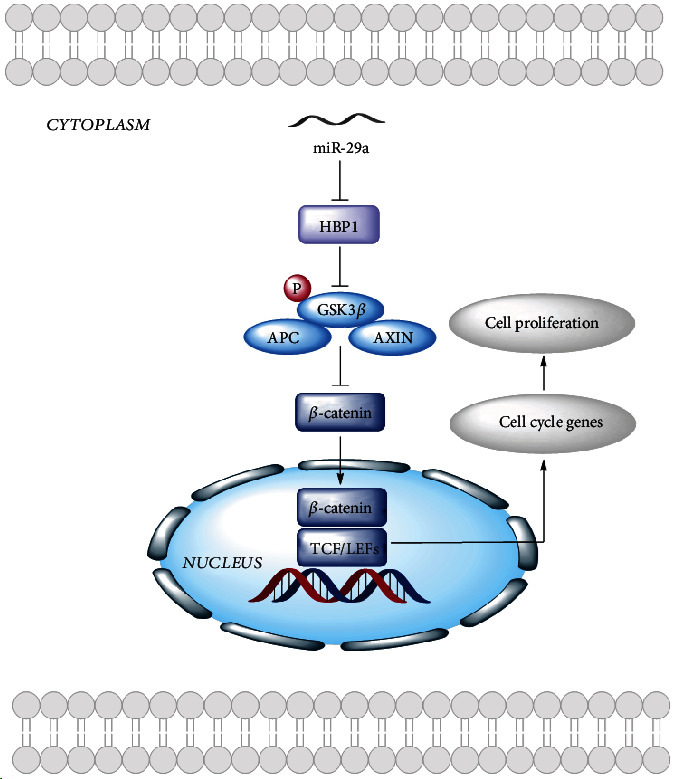
The proposed mechanism of miR-29a-mediated cell proliferation through the Wnt/*β*-catenin pathway.

The miR-29a could directly target HBP1 3′UTR and negatively downregulates its expression level, thereby activating the Wnt/*β*-catenin pathway and causing the promotion in GC proliferation (Figure 8).

## 4. Discussion

POI is a disease characterized by ovarian insufficiency that is detrimental to reproductive health of women [40]. Current therapeutic options include immunomodulation, dehydroepiandrosterone, melatonin, and hormonal therapies [41]. However, hormone therapy is always accompanied by heart disease and endometrium, breast, and ovarian cancer [42, 43]. Therefore, stem cell therapy is proposed [44]. The stem cells are notable for their abilities to self-repair and regenerate [45]. MSC transplantation is considered a successful cell therapy [46]. The human umbilical cord serves as a promising source for collecting MSCs [47]. Collection of MSCs from the human umbilical cord is more convenient and less painful than that from bone marrow stem cells [48]. The MSC-derived exosomes carry miRNAs, messenger RNAs (mRNAs), cytokine, growth factors, and signaling lipids [49]. A previous study has elucidated the critical functions of paracrine effectors in the therapeutic significance of MSC [50]. Exosomes mediate the paracrine effects of stem cell therapy [51]. Previous studies have exhibited the protective effects of exosome-carrying miRNAs. For example, bone MSC-derived exosomes carrying miR-644-5p inhibit the apoptosis of ovarian GC [22]. miR-210 from MSC-derived exosomes inhibits the cardiomyocyte apoptosis [52]. Exosomal miR-34a derived from MSCs inhibits the proliferation of breast carcinoma cells [53].

The ovary is a critical reproductive organ, and a healthy ovary produced sex and gametogenic hormones [54]. The dysfunction of ovarian follicles causes the injury to ovarian physiological functions and reproductive abilities. GCs, critical components of follicles, are associated with follicular evolution, activation, and function [55]. FSH is a hormone that participates in the proliferation of GCs. The decreased sensitivity of FSHR results in the inhibition of follicle development, thereby causing the occurrence of POI [56]. AMH exerts vital roles in follicular recruitment, and AMH level can reflect the reserve of the ovaries [57]. AMH loss contributes to the overrecruitment of follicles, and AMH sheds influence on the sensitivity of primordial follicles to FSHR [58]. In POI serum, the concentration of E_2_ is downregulated [59]. Aberrant level of miR-29a has been observed in POF tissues [26]. miR-29a has been reported to inhibit the apoptosis of various cell types, including cardiomyocytes [60], vascular endothelial cells [61], and spermatogenic cells [62]. In our study, we conducted experiments on the biological effects of human UCMSC-derived exosomes carrying miR-29a and their mechanisms against POI progression. We found that exosomal miR-29a derived from human UCMSCs protects GCs from cisplatin-induced apoptosis in vitro. Furthermore, in vivo, miR-29a upregulation reserved the number of mature follicles and the levels of AMH and E_2_, while it inhibited the FSH level. The above findings showed the protective effects of exosomal miR-29a derived from human UCMSCs against POI-induced ovarian dysfunction.

miRNAs can regulate the expression of targeted mRNAs posttranscriptionally [21]. We herein used bioinformatics database and conducted luciferase reporter assays to predict the target gene of miR-29a. HBP1 caught our attention. HBP1 knockdown protects the mitochondrial function and inhibits the apoptosis of GCs [27]. HBP1 absence leads to reduction in cell apoptosis, thereby enhancing ovarian reserve [63]. We herein discovered that HBP1 elevation counteracted the suppressive effects of miR-29a upregulation on ovarian dysfunction.

As reported, the Wnt/*β*-catenin pathway exerts critical functions in the development of the female reproductive system. The lower Wnt 4 level is positively correlated with poorer ovarian development and fewer healthy follicles [29]. The normal expression of *β*-catenin in ovaries enhances FSH secretion and reduces the GC apoptosis, thereby promoting follicular development [30]. *β*-Catenin escapes degradation and relocates to the nucleus where *β*-catenin cooperates with the TCF/LEF family of transcription factors to activate the pathway [64]. Downregulation of GSK3*β* results in the accumulation of *β*-catenin in the nucleus [65]. We herein found that HBP1 upregulation restored the miR-29a upregulation-induced promotion in the levels of TCF, LEF, and phosphorylated GSK3*β*, inactivating the Wnt/*β*-catenin pathway.

Here, we first discovered that the human UCMSC-derived exosomes carrying miR-29a upregulation promoted the proliferation of GCs and inhibited its apoptosis *in vitro* and *in vivo*; miR-29a upregulation reserved the mature follicles and rescued het decreased levels of AMH and E_2_ and restored the promotion in FSH level. Mechanistically, miR-29a targeted HBP1 and negatively regulated its expression. Furthermore, HBP1 upregulation reversed the suppressive effects of miR-29a upregulation on GC apoptosis and inactivated the Wnt/*β*-catenin pathway.

In summary, the exosomal miR-29a derived from human UCMSCs improves the ovarian function by targeting HBP1 and activating the Wnt/*β*-catenin pathway. Honestly, some limitations should be mentioned. First, this study fails to detect the inflammatory mediators in ovarian tissues. Second, the number of animals in this study is not enough. Despite these limitations, we believe that exosomal miR-29a would be a promising target for POI treatment.

## Figures and Tables

**Table 1:** Sequences of primers used for reverse transcription quantitative PCR.

Gene	Sequence (5′→3′)
miR-29a forward	GCCGAGTAGCACCATCTGAAAT
miR-29a reverse	CTCAACTGGTGTCGTGGA
Adamts12 forward	TCGGTGAAAGGCTTCTGTCG
Adamts12 reverse	TACTGGAAAACGGTCCCTGC
Col9a1 forward	CCACCCCTTTCCTTTGCTTC
Col9a1 reverse	GCAAAGCCATCCGCATCAAT
Col11a1 forward	ACTTGTTGCGTTTCAGCCAG
Col11a1 reverse	ACACAAGAGTGAATTGCAACC
Hbp1 forward	TAATGGCGACGGGTTTGTCG
Hbp1 reverse	GGCAGATTGGGTAGGGTCAC
U6 forward	CTCGCTTCGGCAGCACATATACT
U6 reverse	ACGCTTCACGAATTTGCGTGTC
GAPDH forward	AGGTCGGTGTGAACGGATTTG
GAPDH reverse	GTAGACCATGTAGTTGAGGTCA

## Data Availability

The datasets used during the current study are available from the corresponding author on reasonable request.
